# The TAND checklist: a useful screening tool in children with tuberous sclerosis and neurofibromatosis type 1

**DOI:** 10.1186/s13023-020-01488-4

**Published:** 2020-09-07

**Authors:** Francesca Cervi, Veronica Saletti, Katherine Turner, Angela Peron, Sara Bulgheroni, Matilde Taddei, Francesca La Briola, Maria Paola Canevini, Aglaia Vignoli

**Affiliations:** 1 Epilepsy Center- Child Neuropsychiatry Unit, ASST Santi Paolo Carlo, Department of Health Sciences, University of Milan, Via di Rudinì 8, 20142 Milan, Italy; 2 Developmental Neurology Unit, Fondazione IRCCS Istituto Neurologico Carlo Besta, 20131 Milan, Italy; 3 Department of Health Sciences, University of Milan, Milan, Italy; 4 Department of Pediatrics, Division of Medical Genetics, University of Utah School of Medicine, Salt Lake City, UT USA

**Keywords:** Tuberous sclerosis complex, TSC, Neurofibromatosis 1, NF1, TAND, TAND checklist, Neuropsychiatric, Children

## Abstract

**Background:**

Tuberous Sclerosis Complex (TSC) and Neurofibromatosis type 1 (NF1) are neurocutaneous disorders commonly characterized by neuropsychiatric comorbidities. The TAND (Tuberous Sclerosis Associated Neuropsychiatric Disorders) Checklist is currently used to quickly screen for behavioural, psychiatric, intellectual, academic, neuropsychological and psychosocial manifestations in patients with TSC. We administered the authorized Italian version of the TAND Checklist to the parents of 42 TSC patients and 42 age- and sex-matched NF1 patients, for a total of 84 individuals, aged 4–20 years.

Aims of this study: - to test the overall usability of the TAND Checklist in NF1, −to compare the results between children and adolescents with TSC and NF1, and -to examine the association between neuropsychiatric manifestations and severity of the phenotype in terms of epilepsy severity in the TSC cohort and disease severity according to the modified version of the Riccardi severity scale in the NF1 cohort.

**Results:**

*TSC cohort:* 35.6% had Intellectual Disability (ID), 11.9% Specific Learning Disorders (SLD), 50.0% Attention Deficit Hyperactivity Disorder (ADHD) and 16.6% anxious/mood disorder. 33.3% had a formal diagnosis of Autism Spectrum Disorder (ASD). Paying attention and concentrating (61.9%), impulsivity (54.8%), temper tantrums (54.8%), anxiety (45.2%), overactivity/hyperactivity (40.5%), aggressive outburst (40.5%), absent or delayed onset of language (40.5%), repetitive behaviors (35.7%), academic difficulties (> 40%), deficits in attention (61.9%) and executive skills (50.0%) were the most commonly reported problems.

*NF1 cohort:* 9.5% had ID, 21.4% SLD, 46.6% ADHD, and 33.3% anxious/mood disorder. No one had a diagnosis of ASD. Commonly reported issues were paying attention and concentrating (59.5%), impulsivity (52.4%), anxiety (50.0%), overactivity/hyperactivity (38.1%), temper tantrums (38.1%), academic difficulties (> 40%), deficits in attention (59.5%), and executive skills (38.1%).

*Neuropsychiatric features in TSC* vs *NF1:* Aggressive outburst and ASD features were reported significantly more frequently in TSC than in NF1.

*Neuropsychiatric manifestations and phenotype severity:* Depressed mood, absent or delayed onset of language, repetitive language, difficulties in relationship with peers, repetitive behaviors, spelling, mathematics, dual-tasking, visuo-spatial tasks, executive skills, and getting disoriented were significantly different among TSC patients with different epilepsy severity. No statistically significant differences in the NF1 subgroups were noted for any of the items in the checklist.

**Conclusion:**

The TAND Checklist used for TSC is acceptable and feasible to complete in a clinical setting, and is able to detect the complexity of neuropsychiatric involvement in NF1 as well. NF1 is mainly characterized by an ADHD profile, anxiety problems and SLD, while ASD features are strongly associated with TSC. In conclusion, the TAND Checklist is a useful and feasible screening tool, in both TSC and NF1.

## Background

Tuberous Sclerosis Complex (TSC) and Neurofibromatosis type 1 (NF1) are the two most common neurocutaneous diseases, with an incidence of 1 in 6000 and 1 in 3000 new live births worldwide, respectively [1, 2].

Both are typically diagnosed in early childhood or adolescence, and are lifelong, complex, multisystem and tumor-prone disorders. A wide variety of tissues and organ systems are affected, with large inter and intra-familial clinical variability and age-dependent manifestations [1–3]. Clinical criteria are used to make the diagnosis of TSC and NF1, and can be complemented by molecular testing of the *TSC1/TSC2* and *NF1* genes, respectively [2, 4]. Although characterized by distinctive clinical manifestations, TSC and NF1 share some common characteristics, especially at the neuropsychiatric level.

The most common manifestations in TSC are benign tumors affecting the skin, brain, kidneys, lungs, and heart with possible subsequent organ dysfunction as the normal parenchyma is replaced by a variety of cell types. The prominent neurological issues are subependymal giant cell astrocytomas (SEGAs) and epileptic seizures. The reported prevalence of epilepsy in TSC is 83.6% [5] and remains a major challenge, with more than 60% of the patients having drug refractory seizures [6]. Neurodevelopmental disorders are common: About 50% of affected individuals have normal cognitive function, and the remaining exhibit intellectual disability (ID) of various degrees [7]; at least 30% of school-aged children with TSC are at risk of academic difficulties [8]; almost 50% of the patients have autism spectrum disorder (ASD) and/or attention-deficit–hyperactivity disorder (ADHD) [9, 10]; anxiety and depressive disorders are often identified from early adolescence into adulthood.

Manifestations of NF1 include café-au-lait (CAL) macules, skin-fold freckling (also known as Crowe’s sign), Lisch nodules, cutaneous, subcutaneous and plexiform neurofibromas causing disfigurement and compression of adjacent structures, optic pathway gliomas, skeletal abnormalities, and characteristic malignancies. Additionally, children are prone to cognitive, learning and behavioural disorders [11, 12]. In particular, cognitive deficits include a generalized downshifting Intelligence Quotient (IQ) with cognitive delay in about 4–8% of the patients [4], impairment in visuo-spatial skills in the majority of NF1 children [13, 14], in specific academic domains of reading, spelling, and mathematics in up to 75% [15, 16], and attention problems including ADHD in 60% [17–19]. Difficulties in social functioning with increased rates of ASD are seen in 11% of the patients [20], and mood/anxiety disorders [21] encompassing anxiety, depression, social withdrawal, and somatic complaints have been reported [22].

Patients with TSC and NF1 are followed with disease-specific check-ups to detect medical complications, in order to set up prompt therapeutic interventions. Neuropsychiatric difficulties are common and have a significant impact on the patients’ and families’ quality of life, but are rarely assessed and treated.

In TSC the term TAND (Tuberous Sclerosis Associated Neuropsychiatric Disorders) is used to capture the multidimensional biopsychosocial difficulties of the disease [23]. A specific TAND Checklist has been developed to assess Behavioral, Psychiatric, Intellectual, Academic, Neuropsychological and Psychosocial areas [24]. The purpose of the checklist is to be an easy-to-use, short and accessible tool for every health-care professional in order to assess the neuropsychiatric involvement and to identify patients needing next-step evaluation and treatment [24]. The checklist is freely available online at: https://www.tscinternational.org/wp-content/uploads/2018/11/TAND_checklist-2014.pdf

To date, no psychosocial disease-specific screening tool has been developed to assess NF1 patients [25]. In addition, available recommendations for the diagnosis and clinical management of these aspects have been only recently delineated [26].

Since behavioral, psychiatric, intellectual, academic, neuropsychological and psychosocial areas can be impaired also in NF1 patients, we hypothesized that the TAND Checklist could be useful for screening of neuropsychiatric needs in this population as well and be more broadly applied also to NF1. We therefore administered the TAND Checklist in two homogeneous cohorts of patients with TSC and NF1, and compared the neuropsychiatric manifestations in relation to the severity of their phenotype.

## Methods

The authorized Italian version of the TAND Checklist was administered to 42 consecutively enrolled TSC patients (23 Females and 19 Males, mean age 11.36 ± 4.19 years), followed at the Tuberous Sclerosis Clinic, ASST Santi Paolo e Carlo, Milan, and to 42 NF1 patients matched for age and gender (23 Females and 19 Males, mean age 11.33 ± 4.25 years) from the Neurofibromatosis Clinic, Fondazione IRCCS Istituto Neurologico Carlo Besta, Milan, Italy.

All the 84 patients, aged from 4 to 20 years, met the diagnostic clinical criteria for TSC and NF1 and underwent the specific genetic tests. Brain MRIs and clinical/instrumental disease-specific follow-ups were performed in all individuals. Informed consent was obtained, and this study was approved by the ethics committee of our Institutions.

The Italian version of the TAND Checklist was administered by the same physician (F.C.) to the patients’ parents, during scheduled visits of follow-up assessments or through a telephone interview.

We used the TAND Checklist exactly as is, and only replaced the term “TSC” with “NF1” when needed during the administration. To avoid possible significant discordance between the ratings given by the caregivers and the adolescents and also to have a homogeneous observer/witness ratio for all age groups, we interviewed exclusively the parents. The parent who completed the questionnaire was the caregiver usually involved in the patient’s daily management.

We collected data about cognitive functioning and clinical, neurophysiological and brain imaging characteristics. General development and IQ were respectively evaluated using theGriffiths’ Scales of Infant Development, GMDS-ER and the Wechsler Scales of Intelligence, according to the patients’ age.

Tables 1 and 2 report the clinical characteristics of the sample. To evaluate a possible correlation between the TAND Checklist and the clinical expression of the two diseases, each cohort was divided into subgroups based on the phenotype severity.

**Table 1:** Clinical characteristics of the TSC Cohort

	TSC patients (***n*** = 42)	
Mean age (years) ± SD	11.36 ± 4.19	
Range	4–19	
	**n**	**(&)**
Gender		
Male	19	(45.2%)
Female	23	(54.8%)
Diagnostic criteria met for TSC	42	(100%)
Tubers (brain MRI)	37	(88.1%)
Never had epilepsy	13	(31.0%)
Well controlled epilepsy (seizure free)	16	(38.0%)
Active epilepsy	13	(31.0%)
One or more seizures/day	3	(23.1%)
One or more seizures/week	8	(61.5%)
Sporadic seizures	2	(15.4%)
Seizure types		
Infantile Spasms	4	(13.8%)
Focal seizures	19	(65.5%)
Infantile Spasms + Focal seizures	2	(6.9%)
Generalized seizures	4	(13.8%)
Antiepileptic treatment	28	(66.7%)
Monotherapy	11	(39.3%)
Polytherapy	17	(60.7%)
Neurosurgical treatment for epilepsy	3	(7.1%)
Subependymal giant cell astrocytoma (SEGA)	10	(23.8%)
Neurosurgical treatment for SEGA	2	(20.0%)
SEGA treated with Everolimus	6	(60.0%)
Stable SEGA on brain MRI	2	(20.0%)
Received IQ assessment	42	(100%)
Median IQ ± SD	74.48 ± 28.05	
Range	20–134	
Normal IQ	17	(40.5%)
BIF	10	(23.8%)
Mild ID	4	(9.5%)
Moderate ID	8	(19.0%)
Severe/profound ID	3	(7.1%)
Received formal psychiatric assessment	12	(28.6%)
ASD	4	(33.3%)
ADHD	6	(50.0%)
Anxious/Depressed Disorder	2	(16.6%)
Additional support in school (i.e. IEP)	28	(66.7%)
SLD	5	(11.9%)
Low self-esteem (per parents’ report)	11	(26.2%)
Very high levels of stress in families	16	(38.1%)
Very high levels of stress between parents	18	(42.9%)

**Table 2:** Clinical characteristics of the NF1 Cohort

	NF1 patients (***n*** = 42)	
Mean age (years) ± SD	11.33 ± 4.25	
Range	4–20	
	**n**	**(%)**
Gender		
Male	19	(45.2%)
Female	23	(54.8%)
Clinical criteria met for NF1	42	(100%)
OPG (Optic Pathway Glioma)	14	(33.3%)
Stable OPG on brain MRI	11	(78.6%)
Regressed OPG on brain MRI	2	(14.3%)
Worsened OPG on brain MRI	1	(7.1%)
Other CNS tumors	5	(11.9%)
Neurosurgical treatment	2	(40.0%)
Chemotherapy	1	(20.0%)
Plexiform neurofibromas (NF)	12	(28.6%)
Plexiform NF treated with surgery	2	(16.6%)
Received IQ assessment	42	(100%)
Median IQ ± SD Range	94.32 ± 14.89 59–113	
Normal IQ	33	(78.6%)
BIF	5	(11.9%)
Mild ID	4	(9.5%)
Moderate/severe ID	0	(0.0%)
Received formal psychiatric assessmen	15	(35.7%)
ASD	0	(0.0%)
ADHD	7	(46.6%)
Anxious/Depressed Disorder	5	(33.3%)
Additional support in school (i.e. IEP)	21	(50.0%)
SLD	9	(21.4%)
Low self-esteem (per parents’ report)	15	(35.7%)
Very high levels of stress in families	14	(33.3%)
Very high levels of stress between parents	8	(19.0%)

To date, there is no proposal for classification of patients based on disease severity in TSC. Since one of the major burdens of TSC is the recurrence of seizures, we divided the cohort according to epilepsy severity: no history of seizures, epilepsy that was not active at the time of the evaluation (namely, patients who had been seizure free for the last 6 months), and active epilepsy (epilepsy with variable seizure frequency).

We used a modified version [27] of the Riccardi severity scale [28] and divided the NF1 cohort into: Minimal NF1 (patient has no manifestations that compromise health, but has NF1 features such as CAL macules and freckling only), Mild NF1 (minor medical complications such as mild hypertension, asymptomatic plexiform neurofibroma, or optic glioma), Moderate NF1 (complications that significantly compromise health with orthopaedic complications requiring bracing or surgery, large or symptomatic plexiform and moderate pain), and Severe NF1 (medical history of intractable seizures, severe chronic pain, visual impairment, inoperable tumors, and malignancies). Due to small group size, the moderate and severe subgroups were merged.

### Statistical analysis

Data were analyzed using the Statistical Package for the Social Sciences (SPSS 25 IBM, Chicago, IL, U.S.A.) for Windows.

We used means ± standard deviation (SD) for quantitative variables and absolute counts and frequencies for qualitative variables.

The normality of the distributions of the quantitative variables was verified by applying the Shapiro-Wilk test.

Descriptive analysis of the demographic and clinical characteristics of the patients with NF1 and TSC was performed both on the whole cohort and by stratifying patients according to severity scale. We performed chi-square test for categorized variables and not normally distributed variables, otherwise the Mann-Whitney U test (two groups). We considered a two-tailed *p* value of 0.05 or less statistically significant.

## Results

### Neuropsychiatric manifestations in the TSC and NF1 groups according to phenotype severity

Tables 3 and 4 show the descriptive results of the TAND Checklist in the TSC and NF1 cohorts and relative subgroups according to the phenotype severity.

**Table 3:** Results of the TAND Checklist in the TSC cohort

TAND Features	NO EPILEPSY(n = 13) (30.9%)	Not active EPILEPSY(*n* = 16) (38.1%)	Active EPILEPSY(*n* = 13) (30.9%)	***X***^***2***^	***p*** value
	*n* (%)	*n* (%)	*n* (%)		
**Behavioral level**					
Anxiety	7 (53.8)	7 (43.8)	5 (38.5)	0.644	0.725
**Depressed mood**	**1 (7.7)**	**5 (31.3)**	**0 (0.0)**	**6.389**	**0.041**
Extreme shyness	1 (7.7)	5 (31.3)	2 (15.4)	1.592	0.451
Mood swings	2 (15.4)	5 (31.3)	3 (23.1)	1.001	0.606
Aggressive outbursts	3 (23.1)	7 (43.8)	7 (53.8)	2.669	0.263
Temper tantrums	6 (46.2)	10 (62.5)	7 (53.8)	0.780	0.677
Self-injury	0 (0.0)	3 (18.8)	2 (15.4)	2.622	0.270
**Absent or delayed onset of language**	**2 (15.4)**	**7 (43.8)**	**8 (61.5)**	**5.862**	**0.050**
**Repetitive language**	**0 (0.0)**	**5 (31.3)**	**6 (46.2)**	**7.505**	**0.023**
Poor eye contact	0 (0.0)	4 (25.0)	4 (30.8)	4.585	0.101
**Difficult relationship with peers**	**0 (0.0)**	**7 (43.8)**	**5 (38.5)**	**7.629**	**0.022**
**Repetitive behaviors**	**2 (15.4)**	**4 (25.0)**	**9 (69.2)**	**9.501**	**0.009**
Rigidity	4 (30.8)	5 (31.3)	4 (30.8)	0.001	0.999
Overactivity/hyperactivity	5 (38.5)	6 (37.5)	6 (46.2)	0.255	0.880
Difficulty paying attention or concentrating	5 (38.5)	12 (75.0)	9 (69.2)	4.489	0.106
Restlessness	2 (15.4)	4 (25.0)	4 (30.8)	1.001	0.606
Impulsivity	4 (30.8)	9 (56.3)	10 (76.9)	5.612	0.060
Difficulties with eating	3 (23.1)	5 (31.3)	5 (31.3)	0.721	0.697
Sleep difficulties	2 (15.4)	5 (31.3)	6 (46.2)	2.880	0.237
**Scholastic level**					
Reading	6 (46.2)	6 (37.5)	9 (69.2)	7.008	0.135
Writing	6 (46.2)	8 (50.0)	9 (69.2)	5.663	0.226
**Spelling**	**2 (15.4)**	**6 (37.5)**	**11 (84.6)**	**13.200**	**0.001**
**Mathematics**	**7 (53.8)**	**8 (50.0)**	**11 (84.6)**	**9.609**	**0.048**
**Neuropsychological level**					
Memory	3 (23.1)	4 (25.0)	4 (30.8)	0.218	0.897
Attention	5 (38.5)	11 (68.8)	10 (76.9)	4.591	0.101
**Dual-tasking**	**4 (30.8)**	**10 (62.5)**	**10 (76.9)**	**5.957**	**0.050**
**Visuo-spatial tasks**	**1 (7.7)**	**1 (6.3)**	**6 (46.2)**	**8.981**	**0.011**
**Executive skills**	**3 (23.1)**	**9 (56.3)**	**9 (69.2)**	**5.942**	**0.050**
**Getting disoriented**	**1 (7.7)**	**4 (25.0)**	**7 (53.8)**	**6.946**	**0.031**
**Psychosocial level**					
Low self-esteem	2 (15.4)	7 (43.8)	2 (15.4)	4.123	0.127
Very high levels of stress in families	3 (23.1)	8 (50.0)	5 (38.5)	2.206	0.332
Very high levels of stress between parents	5 (38.5)	8 (50.0)	5 (38.5)	0.538	0.764

**Table 4:** Results of the TAND Checklist applied to the NF1 Cohort, based on clinical severity

Neuropsychiatric manifestations	General Severity NF1 (Riccardi)				
	MINIMAL (*n* = 19)(45.2%)	MILD(*n* = 15)(35.7%)	MODERATESEVERE(*n* = 8)(19.1%)	***X***^***2***^	***p*** value
	*n* (%)	*n* (%)	*n* (%)		
**Behavioral level**					
Anxiety	9 (47.4)	9 (60.0)	3 (37.5)	1.153	0.562
Depressed mood	3 (15.8)	4 (26.7)	2 (25.0)	0.664	0.718
Extreme shyness	4 (21.1)	3 (20.0)	0 (0.0)	1.983	0.371
Mood swings	5 (26.3)	5 (33.3)	1 (12.5)	1.172	0.557
Aggressive outbursts	4 (21.1)	3 (20.0)	1 (12.5)	0.281	0.869
Temper tantrums	9 (47.4)	3 (20.0)	4 (50.0)	3.256	0.196
Self-injury	1 (5.3)	0 (0.0)	0 (0.0)	1.240	0.538
Absent or delayed onset of language	3 (15.8)	3 (20.0)	0 (0.0)	1.768	0.413
Repetitive language	3 (15.8)	1 (6.7)	0 (0.0)	1.850	0.397
Poor eye contact	2 (10.5)	0 (0.0)	0 (0.0)	2.542	0.281
Difficult relationship with peers	3 (15.8)	3 (20.0)	0 (0.0)	1.768	0.413
Repetitive behaviors	1 (5.3)	1 (6.7)	1 (12.5)	0.452	0.798
Rigidity	4 (21.1)	4 (26.7)	3 (37.5)	0.791	0.673
Overactivity/hyperactivity	8 (42.1)	6 (40.0)	2 (25.0)	0.734	0.693
Difficulty paying attention or concentrating	14 (73.7)	6 (40.0)	5 (62.5)	3.984	0.136
Restlessness	4 (21.1)	4 (26.7)	0 (0.0)	2.497	0.287
Impulsivity	12 (63.2)	8 (53.3)	2 (25.0)	3.295	0.193
Difficulties with eating	6 (31.6)	4 (26.7)	3 (37.5)	0.293	0.864
Sleep difficulties	8 (42.1)	3 (20.0)	1 (12.5)	3.258	0.196
**Scholastic level**					
Reading	7 (36.8)	6 (40.0)	1 (12.5)	2.713	0.607
Writing	12 (63.2)	4 (26.7)	3 (37.5)	4.810	0.307
Spelling	7 (36.8)	8 (53.3)	2 (25.0)	1.929	0.381
Mathematics	8 (42.1)	7 (46.7)	5 (62.5)	2.209	0.697
**Neuropsychological level**					
Memory	5 (26.3)	4 (26.7)	3 (37.5)	0.387	0.824
Attention	14 (73.7)	6 (40.0)	5 (62.5)	3.984	0.136
Dual-tasking	7 (36.8)	6 (40.0)	3 (37.5)	0.037	0.982
Visuo-spatial tasks	1 (5.3)	2 (13.3)	1 (12.5)	0.735	0.692
Executive skills	7 (36.8)	5 (33.3)	4 (50.0)	0.638	0.727
Getting disoriented	3 (15.8)	1 (6.7)	0 (0.0)	1.850	0.397
**Psychosocial level**					
Low self-esteem	8 (42.1)	4 (26.7)	3 (37.5)	0.884	0.643
Very high levels of stress in families	6 (31.6)	5 (33.3)	3 (37.5)	0.089	0.957
Very high levels of stress between parents	3 (15.8)	2 (13.3)	3 (37.5)	2.215	0.330

The TSC subgroups were composed of 13/42 (30.9%) individuals with no epilepsy, 16/42 (38.1%) with epilepsy that was not active at the time of the study, and 13/42 (30.9%) with active epilepsy.

The NF1 subgroups were composed of 19/42 (45.2%) patients with minimal disease, 15/42 (35.7%) with mild disease, and 8/42 (19.1%) with moderate/severe disease based on the modified Riccardi scale.

Depressed mood, absent or delayed onset of language, repetitive language, difficulties in relationship with peers, repetitive behaviors, spelling, mathematics, dual-tasking, visuo-spatial tasks, executive skills, and getting disoriented were significantly different among TSC patients with different epilepsy severity. The items that remained constantly highly reported with no statistical difference were: anxiety (53.8–43.8% - 38.5%), temper tantrums (46.2–62.5% - 53.8%), rigidity (30.8–31.3% - 30.8%), overactivity/hyperactivity (38.5–37.5% - 46.2%), difficulties in reading (46.2–37.5% - 69.2%) and writing (46.2–50.0% - 69.2%).

Regarding the NF1 severity subgroups, no statistically significant differences were noted for any of the items in the checklist. The most common behavioral problems were anxiety (47.4–60.0% - 37.5%), overactivity/hyperactivity (42.1–40.0% - 25.0%), temper tantrums (47.4–20.0% – 50.0%), difficulties paying attention or concentrating (73.7–40.0% – 62.5%), impulsivity (63.2–53.3% – 25.0%), sleep difficulties (42.1–20.0% – 12.5%), and attention (73.7–40.0% – 62.5%).

#### Neuropsychiatric manifestations in TSC vs NF1

Table 5 reports the comparison of neuropsychiatric involvement between TSC and NF1 based on the TAND Checklist as reported by the parents.

**Table 5:** Neuropsychiatric features: comparison between NF1 and TSC individuals based on the TAND Checklist

mean age (years) Range	NF1 subjects (***n*** = 42) n (%)	TSC subjects (***n*** = 42) n (%)	***U***	Asymp. Sig. (2-sided) ***p*** value
	11.33 ± 4.25 4–20	11.36 ± 4.19 4–19	880.000	0.986
			***X***^***2***^	
Gender				
Male	19 (45.2)	19 (45.2)	0.000	1.000
Female	23 (54.8)	23 (54.8)	0.000	1.000
**TAND Features**				
**Behavioural level**				
Anxiety	21 (50.0)	19 (45.2)	0.191	0.662
Depressed mood	9 (21.4)	6 (14.3)	0.730	0.393
Extreme shyness	7 (16.7)	8 (19.0)	0.081	0.776
Mood swings	11 (26.2)	10 (23.8)	0.063	0.801
**Aggressive outbursts**	**8 (19.0)**	**17 (40.5)**	**4.613**	**0.032**
Temper tantrums	16 (38.1)	23 (54.8)	2.345	0.126
Self-injury	1 (2.4)	5 (11.9)	2.872	0.090
**Absent or delayedonset of language**	**6 (14.3)**	**17 (40.5)**	**7.244**	**0.007**
**Repetitive language**	**4 (9.5)**	**11 (26.2)**	**3.977**	**0.046**
**Poor eye contact**	**2 (4.8)**	**8 (19.0)**	**4.086**	**0.043**
Difficult relationship with peers	6 (14.3)	12 (28.6)	2.545	0.111
**Repetitive behaviors**	**3 (7.1)**	**15 (35.7)**	**10.182**	**0.001**
Rigidity	11 (26.2)	13 (31.0)	0233	0.629
Overactivity/hyperactivity	16 (38.1)	17 (40.5)	0.050	0.823
Difficulty paying attention or concentrating	25 (59.5)	26 (61.9)	0.050	0.823
Restlessness	8 (19.0)	10 (23.8)	0.283	0.595
Impulsivity	22 (52.4)	23 (54.8)	0.048	0.827
Difficulties with eating	13 (31.0)	13 (31.0)	0.000	1.000
Sleep difficulties	12 (28.6)	13 (31.0)	0.057	0.811
**Scholastic level**				
Reading	14 (33.3)	21 (50.0)	2.400	0.301
Writing	19 (45.2)	23 (54.8)	0.781	0.677
Spelling	17 (40.5)	19 (45.2)	0.194	0.659
Mathematics	20 (47.6)	26 (61.9)	1.732	0.421
**Neuropsychological level**				
Memory	12 (28.6)	11 (26.2)	0.060	0.807
Attention	25 (59.5)	26 (61.9)	0.050	0.823
Dual-tasking	16 (38.1)	24 (57.1)	3.055	0.081
Visuo-spatial tasks	4 (9.5)	8 (19.0)	1.556	0.212
Executive skills	16 (38.1)	21 (50.0)	1.208	0.272
**Getting disoriented**	**4 (9.5)**	**12 (28.6)**	**4.941**	**0.026**
**Psychosocial level**				
Low self-esteem	15 (35.7)	11 (26.2)	0.891	0.345
Very high levels of stress in families	14 (33.3)	16 (38.1)	0.207	0.649
**Very high levels of stress between parents**	**8 (19.0)**	**18 (42.9)**	**5.570**	**0.018**

Significant differences were obtained for aggressive outbursts (*p* = 0.032), absent or delayed onset of language (*p* = 0.007), repetitive language (*p* = 0.046), poor eye contact (*p* = 0.043), repetitive behaviors (*p =* 0.001), getting disoriented (*p* = 0.026), very high level of stress between parents (*p* = 0.018), with the TSC patients more frequently affected.

Anxiety, temper tantrums, rigidity, overactivity/hyperactivity, difficulty paying attention or concentrating and impulsivity, sleep difficulties, reading, writing, spelling and mathematics, attention, executive skills, low self-esteem, very high level of stress in families were very equally reported in patients affected by TSC and NF1.

## Discussion

TSC and NF1 are the most common genetic disorders with cutaneous and neurological involvement. They present many challenges in management due to their heterogeneous presentation and large inter and intra-familial clinical variability. Although their genetic basis and phenotype are different, they are both tumor-prone disorders resulting from the dysregulation of components of the convergent RAS/MAPK and PI3K/AKT/mTOR pathways [29–31].

In both disorders the prevalence of neuropsychiatric problems is relevantly higher than in the general population and impacts quality of life [4, 24]. However, these issues are not always addressed adequately, as physicians are usually more concerned about life-threating complications of both diseases. Moreover, neuropsychiatric evaluation is time-consuming and needs specialized staff. As a result, neuropsychiatric complications may remain underdiagnosed even in expert centers [26].

The TAND Checklist was developed to provide healthcare professionals with a tool to easily screen neuropsychiatric involvement in patients with TSC. The checklist explores the frequency of a wide range of neuropsychiatric manifestations and the multiple dimensions of the involvement on different levels: behavioral, psychiatric, intellectual, academic, neuropsychological and psychosocial [32]. As these aspects can be impaired also in NF1 patients, we hypothesized that the checklist could be useful for screening neuropsychiatric needs in this population as well.

The TAND Checklist showed a wide range of neuropsychiatric issues in our TSC cohort. More than half of the parents reported temper tantrums, difficulty in paying attention and concentrating, impulsivity, scholastic difficulties, attention and executive skills deficits in their children. This profile is in line with the results of TAND data from the large-scale international TOSCA study [33]. In the TSC cohort 69.0% of the patients had a history of epilepsy (38.1% were seizure free at the time of evaluation and the remaining 30.9% had active epilepsy with variable seizure frequency). The lower prevalence of epilepsy in our cohort compared to data in the literature is probably due to the great diversity of patients followed at our TSC Clinic. As a matter of fact, our multidisciplinary TSC Clinic comprises a non-negligible number of affected individuals without neurological problems who were referred to pediatrics, cardiology or genetics for manifestations other than seizures. We found a statistically significant correlation between epilepsy severity and TANDs, except for anxiety, extreme shyness, mood swings, aggressive outbursts, temper tantrums, self-injury, poor eye contact, rigidity, overactivity/hyperactivity, difficult paying attention, restlessness, impulsivity, difficulties with eating, sleep difficulties, academic difficulties (spelling and mathematics) and neuropsychological problems (dual-tasking, visuo-spatial tasks, executive skills, getting disoriented). These last features that are highly reported also in patients with no history of epilepsy, may be considered associated with TSC itself and deserve a deeper consideration both in terms of diagnosis and care in all TSC affected individuals [30]. As we expected, disease-related variables of epilepsy have a significant impact on depressed mood, absent or delayed onset of language, repetitive language and behavior, difficult relationship with peers, and specific neuropsychological domains. The severity of epilepsy, in particular with early onset and poorly controlled seizures, is strongly associated with cognitive impairment and ASD [34–36]. On the other hand, TSC patients without epilepsy did not report any feature associated with ASD. Our findings are in line with the study recently published by Toldo et al. [37], which identified a major impact of early-onset epilepsy on ASD features of TAND in a group of 32 Italian children with TSC, and a higher risk of developing anxious and depressive disorders in individuals with a less severe neurological phenotype.

By administering the TAND Checklist to an age- and gender-matched sample of patients with NF1, we observed difficulties in attention and concentration, impulsivity and anxiety in more than 50% of the patients. Temper tantrums, overactivity/hyperactivity, academic difficulties, executive skill deficits, low self-esteem and very high level of stress in families were reported in more than 30% of children and adolescents with NF1.

Attention problems and ADHD represent well-known behavioral problems in NF1 children as approximately one-third to one-half of children with NF1 fulfill the criteria for ADHD [13]. In line with data from the literature, the TAND Checklist found frequent ADHD-like features: difficulty in paying attention and concentrating in 59.5%, impulsivity in 52.4%, overactivity/hyperactivity in 38.1% and poor attention in 59.5%. These aspects do not show a clear correlation with the disease severity according to the modified version of Riccardi medical severity scale. Of note however, this scale does not include cognitive and behavioral characteristics more directly involved in general adaptive functioning in daily-life [38].

Moreover, regardless of having a comorbid diagnosis of ADHD, children with NF1 show several signs of executive dysfunction compared with typically developing children [39]. Riva et al. [40] found that children with NF1 have specific executive deficits that have an impact on real-life situations. This data is confirmed by our findings from the TAND Checklist applied to NF1, which show the presence of poor executive skills in 38.1% of the patients with NF1.

With regard to emotional and behavioral problems, some studies have evaluated children and adolescents with NF1 through the parents’ compilation of Child Behavior Checklist (CBCL) questionnaires. Rietman et al. [21] showed that of 183 subjects 32% fell in the clinical range, considering Total scores. Graf et al. [22] identified problems predominantly in the internalizing domain of anxiety, depression, social withdrawal and somatic complaints. Studies investigating anxiety in children and adolescents with NF1 have found a higher predisposition to developing an anxiety disorder, but have relied on relatively small sample sizes [41]. In our cohort of NF1 patients 50.0% were reported to have anxiety symptoms, and no statistically significant differences were noted in the three severity subgroups. Only 15 patients with NF1 had received a formal psychiatric assessment, and 7/15 (46.6%) received a diagnosis of ADHD and 5/15 (33.3%) had been diagnosed with anxious or mood disorders. It is therefore possible that the TAND Checklist is useful to identify more NF1 children with dysfunctional behavioral or psychological problems who may benefit from a full behavioral and neuropsychological assessment.

Regarding academic performances, children and adolescents with NF1 commonly perform more poorly at school than how their intellectual abilities would predict [15]. In our sample scholastic difficulties were reported in all domains (reading, writing, spelling and mathematics). Taken together, 41.7% had one or a combination of deficits. Almost all of them received personalized plans and compensatory measures at school, and 21.4% were formally assessed and classified as Specific Learning Disorder.

Taken together, the results of the TAND Checklist applied to NF1 are congruent with the medical literature, are useful to outline a profile of the neuropsychiatric involvement in NF1 and to collect patients’ needs.

In addition, it is noteworthy that parents showed a great interest in this screening tool, asked pertinent questions to the examiner, collaborated with enthusiasm and 21.4% declared the need for a supplementary in-depth analysis of their children’s neuropsychiatric problems, mostly at the behavioral level.

Lastly, we compared the frequencies of the neuropsychiatric manifestations resulting from the checklist in the two conditions. Individuals with TSC were reported to have a greater neuropsychiatric involvement in all the investigated levels.

Cognitive assessment was performed in all TSC and NF1 participants and, as expected, patients with TSC performed lower than patients with NF1. Indeed, the mean IQ in the NF1 cohort was 94 with only 9.5% having mild ID, whereas the mean IQ in the TSC cohort was 74 with 37.7% having various degrees of ID. We found aggressive outburst to be statistically significantly higher in the TSC group. Aggression is common in TSC and is usually associated with stereotyped and repetitive behaviors, low mood, hyperactivity, impulsivity and repetitive use of language, in subjects with intellectual disabilities [42].

We demonstrated statistically significant differences also in the behavioral manifestations of ASD, which are more common in the TSC patients: absent or delayed onset of language, repetitive language, poor eye contact and repetitive behaviors.

It is known that ASD in TSC can be present in 40–50% of the patients [9, 43] being one of the most characteristic disease trait. On the other hand, prevalence rates of clinical ASD symptoms in children with NF1, based on screening instruments, are between 13 to 29% [44, 45] with a statistically significant comorbidity with symptoms of ADHD. A recent study by Eijk et al. [20] used standardized diagnostic methods and found a prevalence of clinical ASD of 10.9%. No one in our NF1 cohort had formal diagnosis of ASD, and the features commonly associated with ASD (such as absent or delayed onset of language, repetitive language, poor eye contact, difficulties in relationship with peers and repetitive behaviors) were reported in less than 15% of the patients.

Individuals with NF1 were recognized to have more difficulties, though not statistically significant, in anxiety, depressed mood and low self-esteem. It can be difficult to investigate these aspects in TSC, given the high rate of ID in this population, and anxiety or depression symptoms can manifest with behavioral changes over time [8]. On the other hand, patients with NF1 have a higher mean IQ and are more aware of their illness.

It is noteworthy that the two samples, despite the differences in IQ levels, had almost identical high rates of ADHD-like symptomatology (overactivity/hyperactivity, difficulty paying attention or concentrating, impulsivity, poor attention and poor executive skills) and of scholastic difficulties (reading, writing, spelling and mathematics). All these features are confirmed to be frequently associated with the disease and deserve a deeper consideration both in terms of diagnosis and care in all TSC and NF1 children.

## Conclusions

This study adds data about the use of the TAND Checklist in the evaluation of patients with TSC and explores the use of this tool in patients with NF1 for the first time.

Our experience confirms the previously reported findings in TSC, and suggests the possibility to extend the use of this tool to screen for neuropsychiatric involvement in other neurological diseases with complex needs.

The TAND Checklist is acceptable and feasible to complete in a clinic setting, and is able to detect the complexity of neuropsychiatric involvement in NF1, as shown by our results. It can be integrated into the routine medical appointments of individuals with NF1 and can produce interpretable and actionable results. The subset of patients who reported a high incidence of issues and is therefore considered at risk for certain neuropsychiatric disorders can be referred for further appropriate assessment and intervention. Furthermore, the checklist can be easily re-administered during follow-up in order to detect the behavioral and psychological changes over time and the efficacy of therapeutic intervention.

Extension studies are warranted, also involving adult individuals, in order to fully characterize the long-term neuro-psychiatric evolution in these disorders. Lastly, investigating the role of IQ in determining the differences observed between the TSC and NF1 cohorts could provide further evidence about the ability of the TAND checklist to discriminate between different clinical samples.

## Data Availability

The data can be obtained from the corresponding author upon request.

## Abbreviations

ADHD: Attention Deficit Hyperactivity Disorder
ASD: Autism Spectrum Disorder
CAL: Café-au-lait
CBCL: Child Behavior Checklist
ID: Intellectual Disability
IQ: Intelligence Quotient
NF1: Neurofibromatosis type 1
SEGAs: Subependymal Giant cell Astrocytomas
SLD: Specific Learning Disorders
TAND: Tuberous Sclerosis Associated Neuropsychiatric Disorders
TOSCA: TuberOus SClerosis registry to increase disease Awareness
TSC: Tuberous Sclerosis Complex
